# Risk factors associated with the development of moderate to severe chronic graft-versus-host disease after non-myeloablative conditioning allogeneic stem cell transplantation in patients with AML or MDS

**DOI:** 10.1007/s13577-019-00297-7

**Published:** 2019-11-15

**Authors:** Laurence M. C. Kok, Laura Bungener, Geertruida H. de Bock, Anouschka Biswana, Geertiena van der Wal, Gustaaf W. van Imhoff, Mar Bellido

**Affiliations:** 1 Department of Hematology, University Medical Center Groningen, University of Groningen, Hanzeplein 1, 9713 GZ Groningen, The Netherlands; 2 Department of Laboratory Medicine, University Medical Center Groningen, University of Groningen, Groningen, The Netherlands; 3 Department of Epidemiology, University Medical Center Groningen, University of Groningen, Groningen, The Netherlands

**Keywords:** Risk factors, Moderate to severe, Chronic graft-versus-host disease, Non-myeloablative, PBSCT

## Abstract

Moderate to severe chronic graft-versus-host disease (cGVHD) is associated with high morbidity, hospital dependency and poor quality of life. In this study, we analyzed a well-defined consecutive series of 98 patients with acute myelogenous leukemia/myelodysplastic syndrome (AML/MDS) who received allogeneic stem cell transplantation with non-myeloablative (NMA) conditioning to determine risk factors associated with the severity of cGVHD. cGVHD was defined according to the 2005 National Institute of Health consensus criteria. Transfusions before transplantation, presence of HLA antibodies, composition of the graft (CD3+, CD19+, CD34+ cells), sibling or matched unrelated donor, female donor to male recipient, CMV serology and the development of acute GVHD (aGVHD), were considered potential risk factors. Multivariate Cox regression analysis identified the number of CD19+ 10^6^/kg (HR 2.79; 95% CI 1.35–5.74), CD3+ 10^6^/kg (HR 2.18; 95% CI 1.04–4.59) infused cells and the presence of patient HLA antibodies before transplantation (HR 2.34; CI 1.11–4.95) as significant risk factors for the development of moderate to severe cGVHD. In summary, we identified in a small, but well-defined cohort, 3 risk factors associated with the severity of cGVHD that should be validated in a larger multi-center study.

**Electronic supplementary material:**

The online version of this article (10.1007/s13577-019-00297-7) contains supplementary material, which is available to authorized users.

## Introduction

AlloSCT is a standard treatment for patients with AML/MDS with high risk of relapse [1–3]. Around 30–70% of transplanted patients who are treated with non-myeloablative conditioning and receive a T-repleted graft collected from peripheral blood develop chronic graft-versus-host disease [4–6]. Moderate to severe cGVHD is associated with high morbidity, hospital dependency and poor quality of life as compared to mild cGVHD [6, 7]. Patients who develop moderate to severe cGVHD are treated with high-dose systemic steroids for months and often years, whereas mild cGVHD can be treated with topical steroids [8, 9]. Long-term steroid treatment impairs immune function and can, therefore, increase the risk of opportunistic infections. Other steroid therapy-related complications include osteoporosis, avascular necrosis, glucose intolerance, cataract, muscle atrophy, hypertension, and disturbance of mood and sleep [10].

Until now, multiple studies have identified risk factors associated with the development of cGVHD [5, 11, 12], but only a few studies have been carried out to identify risk factors associated with the severity of cGVHD [6, 13]. Known risk factors associated with the development of cGVHD independently of the severity of cGVHD are: female donor to male recipient, HLA mismatch, peripheral blood as a source of stem cells, high numbers of infused T cells, recipient age, positive CMV serology and antecedent acute GVHD [5, 11, 12, 14]. Risk factors which are published to be associated with the severity of cGVHD are: transplantation from an immuned female donor to a male recipient, antecedent of aGVHD, CML and NMA conditioning, but most of these factors were identified in the context of bone marrow used as graft source instead of peripheral blood.

Because moderate to severe cGVHD is associated with high morbidity and reduced quality of life and most of the stem cell transplants nowadays use peripheral blood as graft source, it is relevant to identify risk factors that are associated with the development of moderate to severe cGVHD in patients who undergo peripheral blood AlloSCT.

## Materials and methods

### Setting and data collection

This retrospective single center cohort study was conducted at the University Medical Center Groningen (UMCG), the Netherlands. The UMCG is a JACIE accredited tertiary academic hospital that performs all the allogeneic stem cell transplantations in the North of the Netherlands. We identified a homogeneous, consecutive cohort of patients, treated from July 2003 to September 2015 in our center. Patient data were collected from the UMCG transplantation database “ProMise”, and from the UMCG digital patient database. Diagnosis and grading of cGVHD (mild, moderate or severe) were done according to clinical manifestations and the global severity score based on the consensus criteria of the National Institutes of Health (NIH) 2005 [15]. Classification of cGVHD was conducted by the treating physician.

### Treatment protocol

Patients who were included in the study received their AlloSCT between July 16th 2003 and September 4th 2015. Inclusion criteria were: (1) adult patients with AML or MDS who underwent peripheral blood AlloSCT with NMA conditioning, (2) who had received their first AlloSCT without subsequent lymphocyte infusion therapy, (3) had survived at least + 90 days, (4) did not relapse within + 100 days and 5) who developed cGVHD within + 400 days. The last criterion was chosen to identify risk factors that influence the incidence of moderate to severe cGVHD at an early stage and to reduce long-term treatment effects that could bias the outcome of this study. Clinical characteristics of the patients are summarized in Table 1. We defined a control group with the cohort of patients who did not develop cGVHD or who developed only mild cGVHD, versus the cohort of patients who developed moderate to severe cGVHD (Table 2).

**Table 1:** Patient and disease characteristics

	No cGVHD and mild cGVHD (*n* = 60)	Moderate to severe cGVHD (*n* = 38)
Patient sex		
F/m	30/30	14/24
Donor sex		
F/m	23/37	24/14
Patient age		
< 50	15/60 (25%)	9/38 (24%)
≥ 50	45/60 (75%)	29/38 (76%)
Donor age		
< 50	33/60 (55%)	23/38 (60%)
≥ 50	27/60 (45%)	15/38 (40%)
Donor/patient transplant		
♂♂	19/60 (32%)	10/38 (26%)
♂♀	18/60 (30%)	4/38 (10%)
♀♀	12/60 (20%)	10/38 (26%)
♀♂	11/60 (18%)	14/38 (38%)
Transfusions		
< 76	49/58 (84%)	34/36 (94%)
≥ 76	9/58 (16%)	2/36 (6%)
Recipient HLA antibodies positive^a^	6/60 (10%)	11/38 (29%)
CMV positive donor/recipient	52/60 (87%)	34/38 (89%)
CMV positive donor	32/60 (53%)	20/38 (53%)
CMV positive recipient	48/60 (80%)	24/38 (63%)
Risk at diagnosis		
Low risk	1/60 (2%)	1/37 (3%)
Moderate risk	28/60 (47%)	22/37 (59%)
Poor risk/very poor risk	31/60 (51%)	14/37 (38%)
Conditioning		
Flu/TBI	52/60 (92%)	31/38 (82%)
Other	8/60 (8%)	7/38 (18%)
Remission prior to transplantation		
Complete remission	53/60 (88%)	32/38 (84%)
No remission	5/60 (8%)	5/38 (13%)
Persisting aplasia after chemotherapy	2/60 (4%)	1/38 (3%)
Type of transplant		
Sibling	34/60 (57%)	19/38 (50%)
MUD	26/60 (43%)	19/38 (50%)
HLA match		
10/10 MUD	33/60 (55%)	17/38 (45%)
9/10 MUD	1/60 (2%)	2/38 (5%)
Sibling	26/60 (43%)	19/38 (50%)
DPB1		
Match	40/60 (66%)	23/38 (60%)
Permissive	10/60 (17%)	8/38 (21%)
Non-permissive	10/60 (17%)	7/38 (19%)
GVHD prophylaxis		
CsA/MMF	56/60 (94%)	32/38 (8%)
Other	4/60 (7%)	6/38 (16%)
Infused cells	Median (range)	Median (range)
CD3+ 10^6^/kg	246.8 (80.0–573.0)	303.6 (140.2–642.6)
CD19+ 10^6^/kg	55.6 (12.0–131.3)	70.4 (18.0–333.5)
CD34+ 10^6^/kg	6.6 (2.2–18.5)	6.4 (2.6–13.5)

*GVHD* graft-versus-host disease, *HLA* human leukocyte antigen, *CMV* cytomegalovirus, *Flu* fludarabine, *TBI* total body irradiation, *MUD* matched unrelated donor, *CsA* cyclosporine, *MMF* mycophenolate mofetil

^a^HLA antibodies found prior to transplantation in the serum

**Table 2:** cGVHD distribution

	No cGVHD and mild cGVHD (*n* = 60)	Moderate to severe cGVHD (*n* = 38)
Acute GVHD		
Grade I	5/60 (8%)	4/38 (10%)
Grade II–IV	18/60 (30%)	8/38 (20%)
Chronic GVHD		
Mild	27/60 (45%)	**–**
Moderate	–	27/38 (71%)
Severe	–	11/38 (29%)
Cause of death		
Transplantation-related mortality	6/60 (10%)	2/38 (5%)
Relapse	15/60 (25%)	2/38 (5%)
Other	2/60 (3%)	–

Chemotherapy regimens before transplantation consisted of induction with idarubicin (12 mg/m^2^ iv) and cytarabine (200 mg/m^2^ iv) with or without G-CSF (5 ug/kg sc), followed by consolidation with amsacrine (120 mg/m^2^ iv) and cytarabine (1000 mg/m^2^ iv).

Non-myeloablative conditioning consisted of fludarabine (30 mg/m^2^ iv days -4 until -2) and total body irradiation (2 Gy, day -1) in 85% of patients (appendix Table 1). Prophylaxis for GVHD consisted of cyclosporine (CsA) started on day -3 (twice daily 5 mg/kg) and mycophenolate mofetil (MMF) started on day 0 (twice daily 15 mg/kg oral) [16]. MMF was stopped at day + 28 or gradually tapered after day + 40 depending on the type of donor (SIB or MUD, respectively) and CsA was gradually tapered after day + 100 in the absence of GVHD. Acute GVHD grade II was treated as first line with systemic prednisolone 1–2 mg/kg/day and therapeutic doses of CsA. Chronic GVHD was treated as first line with local therapy as mild (0.1% triamcinolone cream, tacrolimus cream, dexamethasone 0.01% suspension) or with prolonged schedule of systemic prednisolone (1 mg/kg/day) progressively tapered over 1 year, in combination with a calcineurin inhibitor in case of moderate to severe cGVHD.

All patients were transplanted with a 10/10 matched SIB or MUD, except 4 patients. One of these 4 patients was transplanted with a 9/10 HLA-A mismatched SIB (patient homozygous on A locus) and three patients were transplanted with a 9/10 mismatched MUD (2 A mismatched and 1 DQB1 mismatched). None of the patients transplanted with a mismatched donor had HLA antibodies against the mismatched locus. Matching was performed using standard high-resolution typing of HLA-A, B, C, DRB1, and DQB1. All patients were typed for HLA-DPB1 and the DPB1 T Cell epitope algorithm was used to determine permissiveness of the combinations [17].

The number of cells in the graft (CD3+, CD19+, CD34+) was determined using fluorescence-activated cell sorting. Patient serum samples collected before transplantation were examined on HLA class I and class II IgG antibody reactivity using complement-dependent cytotoxicity and/or bead array (Lifescreen de luxe or LSA, Immucor). HLA antibody positivity was determined according to the manufacturer.

### Risk factors

Three categories of risk factors were analyzed for association with the development of moderate to severe cGVHD: (1) pre-transplantation factors: recipient: age, number of transfusions before transplantation, presence of HLA IgG antibodies and HLA-DPB1; (2) donor-related factors: age, composition of the graft (CD3+, CD19+, CD34+ cells), type of donor, female donor to male recipient and positive CMV serology; (3) post-transplantation factor: development of aGVHD.

### Statistical analysis

To describe patients and their disease, patients were stratified on the main outcome: no to mild cGVHD versus moderate to severe cGVHD. Time started at the date of transplantation and ended when moderate or severe cGVHD occurred. Death or relapse was a censoring event unless moderate or severe cGVHD was present before death or relapse. A cutoff Q75 was selected to quantify the effect of the donor-infused cells on the development of moderate to severe cGVHD. Univariate Cox regression analyses were performed to estimate time to the development of moderate to severe cGVHD. In this way, hazard ratios (HRs) and 95% confidence intervals (95% Cis) were provided. In the multivariable stepped forward Cox Regression analyses, all independent variables that contributed statistically significantly to the univariate analysis (*P* < 0.20) were entered. To correct for any confounding effect from SIB or MUD transplantation on the outcome, the type of transplant source was added to the multivariate Cox regression model. Risk factors with a *P* value < 0.05 were considered statistically significant. Comparing clinical characteristics between patients according to the identified risk factors were performed using the independent *t* test and Fisher’s exact test. Relapse and treatment-related mortality were calculated using Kaplan–Meier and comparisons were done by log-rank test. Analyses were performed in SPSS 22.

## Results

**Fig. 1:**
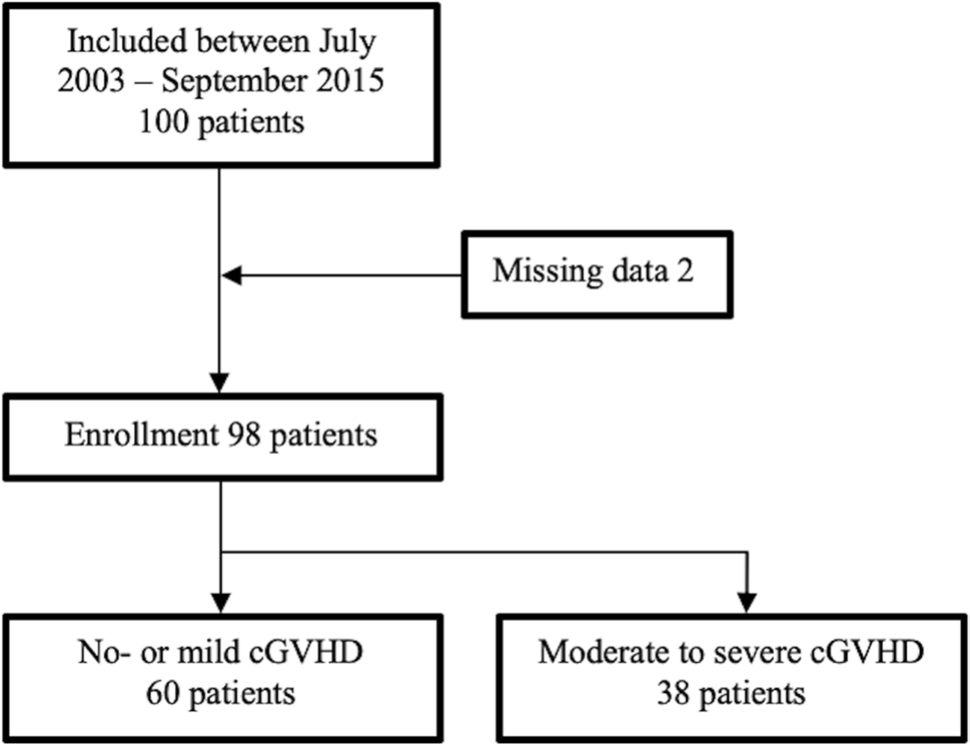
Cohort selection and patient distribution according to no or mild versus moderate to severe cGVHD

A total of 100 consecutive patients fulfilled the inclusion criteria for this study. Two patients were excluded from this cohort due to missing data (Fig. 1). The final study cohort comprised 98 patients, 85 with AML and 13 with MDS. Median patient age at the time of transplantation was 57 years (range 24–74) and median donor age was 45 years (range 19–71). Clinical characteristics of the patients are summarized in Table 1.

**Fig. 2:**
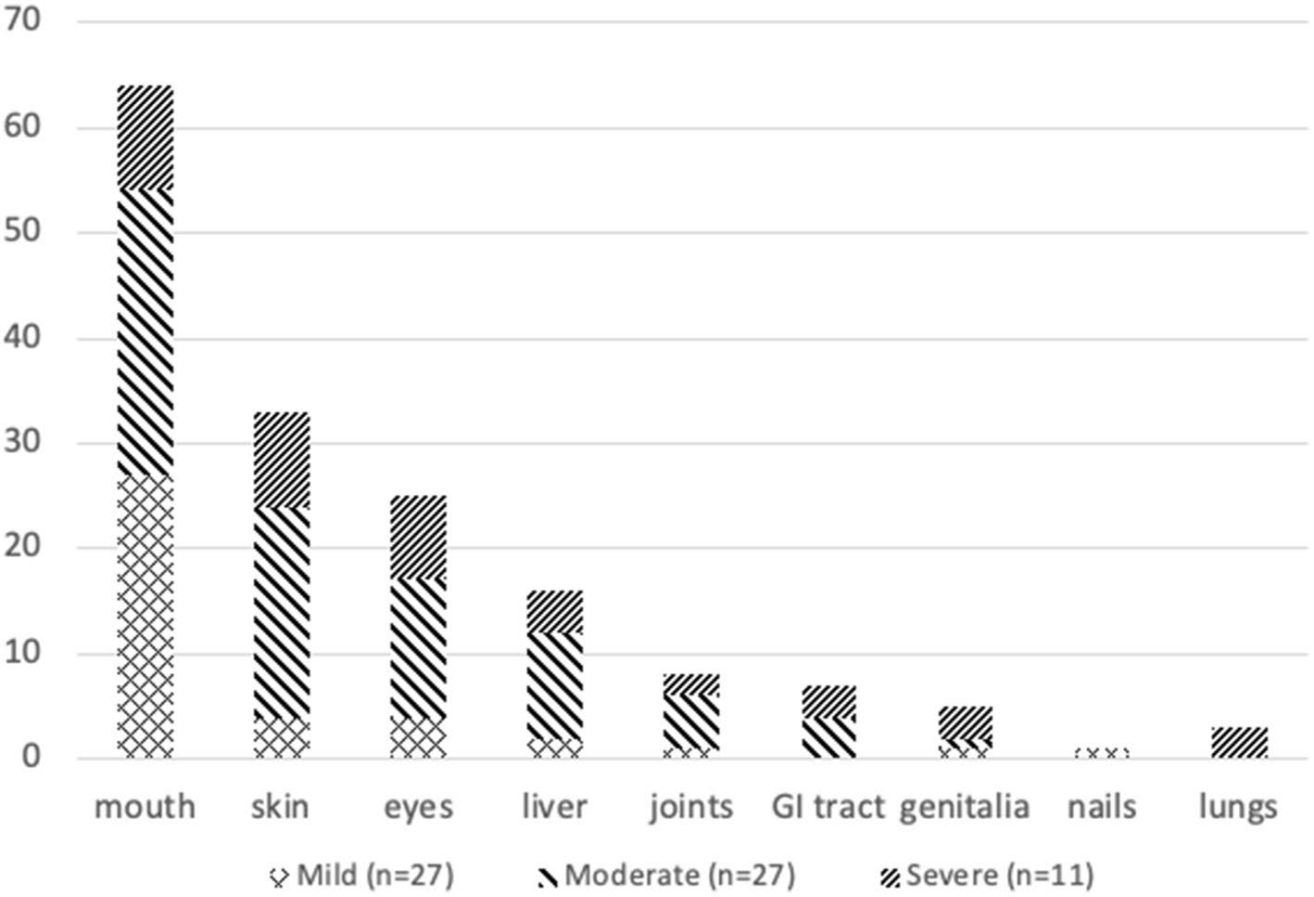
Distribution of cGVHD severity according to organ involvement

In our cohort, 65/98 (67%) patients developed cGVHD, 27 patients developed mild cGVHD and 38 patients developed moderate to severe cGVHD. The median time from transplantation to onset of mild cGVHD was 208 days (26–398). Onset of mild cGVHD was: de novo (no prior aGVHD) in 17 (63%), quiescent (prior aGVHD, but not currently active) in 3 (11%) patients, and progressive (progression from aGVHD to cGVHD) in 7 (26%) patients. The most frequent organ involvement in patients who developed mild cGVHD was mouth (100%), followed by eyes (15%) and skin (15%). Overall cGVHD severity distribution is described in Table 2. The median time from transplantation to onset of moderate to severe cGVHD was 208 days (54–380). Onset of moderate to severe cGVHD was: de novo in 26 (68%) patients, quiescent in 2 (5%) patients, and progressive in 10 (27%) patients. The most frequent organ involvement in patients who developed moderate to severe cGVHD was mouth in 37 (97%) patients, followed by skin in 29 (76%) and eyes in 21 (55%) patients. Other affected organs were liver in 37%, joints in 18%, gastrointestinal tract (GI) in 18%, genital/urologic tract in 10%, and lungs in 8% of the patients. The median number of organs involved in moderate to severe cGVHD was 3 per patient. The organ distribution according to severity of cGVHD in both groups is shown in Fig. 2.

### Pre-transplantation factors associated with cGVHD

In the univariate analysis, we identified the following variables as risk factors for the development of moderate to severe cGVHD: presence of HLA antibodies in the patient before transplantation, graft composition with CD3+ cells ≥ 325 10^6^/kg or CD19+ cells ≥ 82 10^6^/kg. Multivariate Cox regression analysis confirmed HLA antibodies (*P* = 0.03, HR = 2.34, CI 95% 1.11–4.95), CD3+ cells in the graft ≥ 325 10^6^/kg (*P* = 0.04, HR 2.18, CI 95% 1.04–4.59) and CD19+ cells in the graft ≥ 82 10^6^/kg (*P* < 0.01, HR = 2.79, CI 95% 1.35–5.74) as independent risk factors associated with the development of moderate to severe cGVHD (Table 3). Other factors including type of donor were not significantly associated with the severity of cGVHD in this cohort.

**Table 3:** Variables related to the development of moderate to severe cGVHD

Risk factors	Univariate			Multivariate		
	HR	95% CI	*P*	HR	95% CI	*P*
Patient age						
< 50 years	1					
≥ 50 years	1.18	0.55–2.50	0.662			
Donor age						
< 50 years	1					
≥ 50 years	0.874	0.45–1.67	0.686			
Blood transfusions						
< 76	1					
≥ 76	0.38	0.09–1.61	0.192			
HLA antibodies in the recipient^a^						
HLA antibodies negative	1					
HLA antibodies positive	**2.49**	**1.22–5.08**	**0.012**	**2.34**	**1.11–4.95**	**0.026**
Donor/recipient CMV status						
Matched	1					
Mismatched	1.04	0.37–2.94	0.935			
Donor/recipient gender						
Matched	1					
Female to male	1.80	0.93–3.48	0.081			
Type of donor						
SIB	1					
MUD	1.36	0.72–2.59	0.342			
DPB1						
Match	1					
Permissive	1.13	0.51–2.53	0.768			
Non-permissive	1.40	0.60–3.28	0.441			
Permissive/non-permissive	1.24	0.65–2.38	0.519			
Infused cells in the recipient:						
CD3 + 10^6^/kg < 325	1					
CD3 + 10^6^/kg ≥ 325	**3.06**	**1.56–6.00**	**0.001**	**2.18**	**1.04–4.59**	**0.040**
CD19 + 10^6^/kg < 82	1					
CD19 + 10^6^/kg≥ 82	**3.77**	**1.93–7.37**	**0.000**	**2.79**	**1.35–5.74**	**0.005**
CD34 + 10^6^/kg < 8.6	1					
CD34 + 10^6^/kg ≥ 8.6	1.47	0.73–2.97	0.281			
GVHD						
No acute GVHD	1					
Acute GVHD	0.84	0.42–1.66	0.612			

Total of infused cells boundaries were determined based on Q75 for all cells

*HLA* human leukocyte antigen, *CMV* cytomegalovirus, *GVHD* graft-versus-host disease

^a^HLA antibodies found prior to transplantation in the serum

Bold values indicate that statistical significance *P* < 0.05)

Comparing the clinical characteristics between patients according to the identified risk factors, there were no differences among groups. In the group of patients with positive vs negative pre-transplant HLA antibodies (17 vs 81 patients), median patient age was 54 (35–68) vs 56 years (24–74) (*t*(96) = 1,17, *P* = 0.24), median donor age was 40 (20–66) vs 48 years (19–71) (*t*(96) = 1.88, *P* = 0.06), 76% vs 86% received fludarabine/TBI as conditioning (*P* = 0.24) and 90% in both groups received CsA/MMF as immunosuppression (*P* = 0.54). Nevertheless, 11 (65%) vs 27 (33%) patients developed moderate to severe cGVHD. The HLA antibodies detected in these 17 patients were directed against HLA class I in 6 patients, against class HLA class II in 2 patients and against HLA class I and II in 9 patients. None of the patients with HLA antibodies had donor-specific antibodies.

Comparing the group of patients who received grafts with ≥ 325 vs < 325 10^6^/kg CD3 + cells (24 vs 73 patients), median patient age was 58 (44–69) vs 55 years (24–74) (*t*(95) = − 1.16, *P* = 0.24), median donor age was 44 (20–69) vs 45 years (19–71) (*t*(33) = 0.28, *P* = 0.78), 75% vs 88% received fludarabine/TBI as conditioning (*P* = 0.19) and 83% vs 91% received CsA/MMF as immunosuppression (*P* = 0.26). Nevertheless, 15 (63%) vs 22 (30%) patients developed moderate to severe cGVHD.

Comparing the group of patients who received grafts with ≥ 82 vs < 82 10^6^/kg CD19 + cells (24 vs 71 patients), median patient age was 59 (44–69) vs 56 years (24–74) (*t*(93) = − 1.41, *P* = 0.16), median donor age was 47 (20–67) vs 45 years (19–71) (*t*(93) = 0.071, *P* = 0.48), 79% vs 86% received fludarabine/TBI as conditioning (*P* = 0.31) and 96% vs 87% received CsA/MMF as immunosuppression (*P* = 0.22). Nevertheless, 16 (67%) vs 20 (28%) patients developed moderate to severe cGVHD.

Comparing treatment-related mortality (TRM) 3 years after AlloSCT between patients in the control group versus the group with moderate to severe cGVHD, we saw no significant difference (15% vs 5%, *P* = 0.20). Transplantation-related causes of death are described in Table 4.

**Table 4:** Transplantation-related causes of mortality

	No cGVHD	Mild cGVHD	Moderate to severe cGVHD
Sepsis	1	1	1
Progressive encephalopathy	1	–	–
Multiple viral/bacterial infections	1	–	–
Treatment-resistant aGVHD	1	–	–
Multi-organ failure	1	–	1

Comparing the occurrence of relapse rate (RR) 3 years after AlloSCT, we found a significantly higher cumulative RR in patients in the control group versus patients with moderate to severe cGVHD (30% vs 7%, *P* < 0.01). Six (10%) patients in the control group relapsed within 6 months with no acute or mild cGVHD, 3/60 (6%) patients relapsed within 6 months with previous aGVHD, 1/60 (2%) relapsed within 6 months with previous acute and mild cGVHD, the other (8%) patients relapsed after 6 months. In the moderate to severe group, two patients relapsed, after 11 months and 3 years with moderate cGVHD. In our study, the group “no cGVHD and mild cGVHD” has a significantly higher relapse rate after 3 months and within 1 year after ALLOSCT compared with the group who developed moderate to severe cGVHD. The high relapse rate in this group explains the higher mortality rate in the group “no cGVHD and mild cGVHD” versus “moderate to severe cGVHD” (appendix Table 2). If we analyzed which factors could explain this difference, we know that the distribution of patients according to AML/MDS risk status by diagnosis and according to remission status before ALLOSCT (low—and intermediate risk versus poor—and very poor risk) did not differ between these 2 groups. Moreover, the conditioning regimen, immunosuppression regimen and the type of donor did not differ between these 2 groups. Thus, the factors related with a high percentage of relapses in the group with no-GVHD may be related to early immunological factors (as activation of donor T cells, migration of donor immune cells into target organs and thymic injury) which avoid tolerance and therefore recognize residual leukemic cells avoiding relapses in patients who develop moderate to severe cGVHD [18].

## Discussion

In this well-defined unselected series of patients diagnosed with AML and/or MDS who consecutively received a peripheral blood T-cell repleted NMA AlloSCT, moderate or severe chronic graft-versus-host disease was associated with high morbidity, hospital dependency and poor quality of life as compared to mild cGVHD [6, 7]. The goal of this study was to identify risk factors associated with severity, i.e., with the development of moderate to severe cGVHD in this population.

We identified 3 independent risk factors associated with the development of moderate to severe cGVHD: HLA antibodies in patient’s serum before transplantation, CD19+ ≥ 82 10^6^/kg cells and/or CD3+ ≥ 325 10^6^/kg cells in the graft.

Our results on the correlation of HLA antibodies with the severity of cGVHD are in accordance with a previously reported study [19]. Pan et al. described a correlation between the presence in patients’ serum of antibodies against HLA before or in the 1st month after transplantation and cGVHD. They studied a cohort of patients diagnosed with hematological malignancies who received a BM/PBSCT with myeloablative conditioning. They found a higher rate of extensive cGVHD in the group with HLA antibodies in comparison to the group of patients without HLA antibodies prior to transplantation [19]. In our study, the HLA antibody status before transplantation was associated with the development of moderate to severe cGVHD. We know that pre-formed HLA antibodies prior to AlloSCT can be unaffected by standard transplantation conditioning regimens [20]. Broad sensitization against many HLA antigens can occur when the immune system is only exposed to a single non-self HLA antigen, resulting in HLA antibodies that can react to more than one antigen (cross-reactivity) [21]. In a patient receiving allogeneic cells followed by a changing very active immune system, HLA antibody cross-reactivity to non-self or auto-antigens could stimulate auto-inflammatory reactions. Having identified HLA antibodies in patients before AlloSCT as risk factor for moderate to severe cGVHD, we hypothesize that the presence of HLA antibodies may indicate a state of high immune reactivity in patients, increasing the probability of triggering a more severe cGVHD.

We also identified the amount of infused CD19+ cells as a risk factor for the development of moderate to severe cGVHD. Recent studies demonstrated that B cells play an important role in the complex immuno-pathophysiology of cGVHD having an effector function, generating alloantibodies and producing transforming growth factor beta [22–25]. Patients with cGVHD have high levels of B-cell-activating factor, present with increased survival of alloreactive- and auto-reactive B cells, and subvert the development of B-cell tolerance by attenuating B-cell receptor-triggered apoptosis of newly created polyreactive B cells [23, 24]. Moreover, a subtype of B cells (CD19+ CD21 low) has been found as an expanded population with features of exhaustion in patients with cGVHD and they have been correlated with severity of cGVHD [26]. These articles support the role of CD19+ cells as a parameter of severity in patients with cGVHD.

The correlation between the number of T cells in the graft with the incidence of moderate to severe cGVHD is in accordance with previously published studies [14, 27, 28]. These reports show a lower incidence of cGVHD in patients transplanted with BM as source of stem cells as compared to PB stem cells. Typically, PBSC grafts obtained using G-CSF for PBSC mobilization contain more T cells than BM grafts, resulting in a higher risk to develop GVHD [14, 27–30].

Our study has limitations that can explain why other expected risk factors as CMV serostatus and recipient gender were not found associated with the development of moderate to severe cGVHD [6, 31]. In our cohort, we did not focus on the development of global cGVHD, but we focussed on severity. A role of CMV serostatus as a risk factor for the development of moderate to severe cGVHD was not found. This could be explained by the fact that almost all donor/recipients in our cohort had a positive CMV serostatus and therefore had no discriminating value. For female donor to male recipient transplantations, there was a trend towards a higher chance for moderate to severe cGVHD, but this did not reach statistical significance. We also did not find a role of previous aGVHD as a predictor for the development of moderate to severe cGVHD, as opposed to other studies [5, 6]. A possible explanation may be early RR and TRM that occurred in the control group, resulting in a relatively high mortality rate within 6 months after transplantation. Nevertheless, the association of the identified risk factors with severity of cGVHD has biological support and opens up the opportunity to be validated in a multi-center study.

In conclusion, we identified HLA antibodies in patients and number of CD19 + and/or CD3 + cells in the graft to be associated with the development of moderate to severe cGVHD in patients with AML or MDS who received a NMA AlloPBSCT. Our results should be confirmed in a larger multi-center cohort of patients to confirm clinical significance.

## Electronic supplementary material

Below is the link to the electronic supplementary material.

Supplementary material 1 (DOCX 14 kb)

Supplementary material 2 (DOCX 14 kb)
