# Supplementary material for: Risk factors associated with the development of moderate to severe chronic graft-versus-host disease after non-myeloablative conditioning allogeneic stem cell transplantation in patients with AML or MDS
Source: Hum Cell. 2019 Nov 15;33(1):243–51. doi: 10.1007/s13577-019-00297-7 (PMC6965489; doi:10.1007/s13577-019-00297-7)
Supplement: Supplementary file 2 — Supplementary material 2 (DOCX 14 kb) [file 13577_2019_297_MOESM2_ESM.docx]

**Risk factors associated with the development of moderate to severe chronic graft-versus-host disease after non-myeloablative conditioning allogeneic stem cell transplantation in patients with AML or MDS.**

**Journal name:** Human Cell

**Authors**

Laurence MC Kok^1^, Laura Bungener^2^, Geertruida H de Bock^3^, Anouschka Biswana^1^, Geertiena van der Wal^1^, Gustaaf W van Imhoff^1^, Mar Bellido^1^.

Affiliations

^1^Department of Hematology, University of Groningen, University Medical Center Groningen, Groningen, The Netherlands

^2^Department of Laboratory Medicine, University of Groningen, University Medical Center Groningen, Groningen, The Netherlands

^3^Department of Epidemiology, University of Groningen, University Medical Center Groningen, Groningen, The Netherlands

**Corresponding author:** Laurence Kok

E-mail: [l.m.c.kok@umcg.nl](mailto:l.m.c.kok@umcg.nl)

| ***Appendix table 2. Cause of death 1-year post AlloSCT*** | | | |
| --- | --- | --- | --- |
|  | **No cGVHD** | **Mild cGVHD** | **Moderate to severe cGVHD** |
| Relapse | 9 | 2 | - |
| Sepsis | 1 | - | 1 |
| Progressive encephalopathy | 1 | - | - |
| Multiple viral/bacterial infections | 1 | - | - |
| Treatment resistant aGVHD | 1 | - | - |
| Multi-organ failure | 1 | - | - |
